# Bone Deposits in Carcinoma of the Breast

**Published:** 1910-07-23

**Authors:** 


### SPECIAL ARTICLES.

### BONE DEPOSITS IN CARCINOMA OF THE BREAST.

Secondary deposits of carcinoma in the bones are of interest for two reasons. First, why should they be so relatively common in some and so very rare in other varieties of cancer? Secondly, how do the metastatic growths reach their resting-place in the bone marrow?

Of all the numerous forms of primary carcinoma, cancer of the breast is the only one in which secondary carcinomatosis of the bones is anything but a pathological curiosity. In fact, in the case of very many of the carcinomata, a metastasis in bone has never yet been recorded.

In mammary cancer the bones most often affected are the sternum, humerus, femur, and spine. The frequency with which they are found to be involved may be said to vary directly with the care devoted to post-mortem investigation. The statistics of one observer will show a very low percentage, while the figures given by another more interested in this aspect of pathology will furnish a surprisingly high proportion of cases. A reason for this is not far to seek. Almost the only clinical feature which attracts attention to the bones in cases of advanced mammary cancer is the occurrence of so-called "spontaneous fracture," a condition which results only when the major portion of the thickness of a bone has been replaced by growth. Failing this, nothing short of actual inspection of the medullary cavity after death will reveal the change the bony tissue has undergone.

### Some Diagnostic Methods.

We may, however, except those cases in which systematic *x*-ray examination of the bones at short intervals has revealed a progressive alteration in the density of the skiagraphic shadow—a very valuable method of investigating the onset and development of metastatic growth. Apart from this, however, in the absence of spontaneous fracture, it is obvious that unless very careful post-mortem examination is carried out many cases must escape observation.

A rough and ready method favoured by many pathologists is the application of considerable force to each of the four limbs when the body is on the post-mortem table, in the expectation that fractures will be produced if any metastatic growths are present.

A neater and more effective method consists in attempting to thrust a stout sharp-pointed needle into the bones in various situations. A deposit of growth of any size is almost certain to have come so close to the surface at some point or other as to lead to such thinning of the overlying bone that it is easily penetrated by the needle. Any soft place so discovered, as in the case of any fracture produced spontaneously or by post-mortem violence, should then be cut down upon and investigated. These methods, necessarily crude, leave many loopholes; but many considerations forbid detailed dissection and inspection of the various bones that are liable to metastasis. However, if careful methods be carried out the presence of bony metastasis in cancer of the breast will be found to be much more frequent than is commonly supposed.

### The Embolic Hypothesis.

A consideration of the manner in which these deposits reach the bone opens one of the most interesting chapters in the history of surgical pathology. For very many years it was held, in a vague and general sort of way, that the only possible route by which cancer cells could reach the medullary cavity of a bone was by a process of embolism *via* the blood vascular system. In 1889 Mr. Stephen Paget took the subject in hand and emphasised some of the difficulties in this hypothesis. For after pointing out that embolism is necessarily an impartial process to which all organs and tissues are equally liable, he brought forward figures illustrating the fact that whereas some bones are not infrequently invaded by cancer others invariably escape. In order to explain this phenomenon he was forced to the conclusion that tissue-predisposition is an important factor in determining whether a given embolus shall survive and grow or be destroyed, or at least rendered incapable of development.

But given this predisposition another difficulty presented itself. Why is cancer of the breast so frequently associated with bone deposits, and other forms of cancer—as, for example, cancer of the stomach—never? In order to account for this another hypothesis was necessary, and one was driven to the further assumption that the tissue-predisposition of the various organs and tissues varies according to the particular situation in which the primary growth originates.

### Handley's Theories.

In the light of our fuller and more recent knowledge this assumption of tissue-predisposition and its variation in relation to different primary growths appears very unreal and very unconvincing; none the less the embolic origin of metastatic growth in bone is a view still believed by some pathologists. The convincing work of Sampson Handley has thrown an entirely new light upon this process, and it is probable that in a little while his views will find general acceptance, not only as regards metastases in breast cancer but in the majority of cases of secondary growth, no matter what the primary seat of origin.

Briefly his theory is that cancer of the breast spreads almost entirely by the lymphatics. He has shown that the first step in the process is the invasion by cancer cells of the lymphatic plexus lying in the deep fascia, and that by continuous growth they in process of time permeate the lymphatic channels and fill their many ramifications.

He has shown that this process goes on in a centrifugal manner, with the primary growth as the centre of an ever-increasing circle of invaded lymph channels. These channels with their wide anastomoses extend wherever the deep fascia extends. The humerus near the insertion of the deltoid and the femur just below the great trochanter have prolongations of the deep fascia attached to them. And these are the points at which secondary growth so commonly manifests itself. The conclusion is inevitable that growth extends into these bones by means of the lymphatic channels we know to be present in the fascia.

Lower than the femur in the leg and lower than the humerus in the arm metastasis in bone is unknown in cancer of the breast; and below these points skin nodules are also invariably absent. An explanation for this is afforded by the centrifugal manner in which permeation takes place. Patients do not live long enough to permit of the centrifugal permeation of parts so far distant from the primary growth.

### A Probable Explanation.

From the foregoing we have some indication of the reason why it is only in cancer of the breast that bony deposits are so frequently met with. It is owing to the fact that the breast has a close and intimate relationship with the subcutaneous tissues and the deep fascial plexus of lymphatics in a degree not possessed by any other organ.

This view of the mode in which extension of mammary cancer takes place has much more than a mere academic interest, important only to pathologists. For presuming its truth we have an entirely logical and scientific basis upon which to plan our operations for the removal of the cancerous breast. Formerly it was the general rule that no matter where the primary growth the breast and all its connections should be removed. But now it is obvious that the exact position of the primary growth must be carefully considered and that the parts removed must represent a circle with the growth as the centre of that circle, for only by operating in this manner is it possible to remove an adequate margin of the lymphatic-bearing fascia all round the growth. As Handley says in his book on "Cancer of the Breast," the removal of the breast must simply be regarded as a necessary incident of the operation, the adequate removal of all possibly permeated lymphatics being the first consideration.

